# An intravaginal ring for real-time evaluation of adherence to therapy

**DOI:** 10.1371/journal.pone.0174729

**Published:** 2017-04-06

**Authors:** John A. Moss, Marc M. Baum, Jeremiah T. Easley, Darren M. Cox, Thomas J. Smith

**Affiliations:** 1 Department of Chemistry, Oak Crest Institute of Science, Monrovia, California, United States of America; 2 Preclinical Surgical Research Laboratory, Colorado State University, Fort Collins, Colorado, United States of America; 3 Auritec Pharmaceuticals, Inc., Pasadena, California, United States of America; Laurentian, CANADA

## Abstract

Two recent Phase III clinical trials to investigate an intravaginal ring for preventing HIV infection demonstrated that adherence to prescribed device use was a primary driver of efficacy. Surrogate methods for determining adherence in the studies were limited in their inability to monitor temporal patterns of use and allow deconvolution of the effects of adherence and device efficacy on HIV infection rates. To address this issue, we have developed functionality in an intravaginal ring to continuously monitor when the device is being used and maintain a log of adherence that can be accessed by clinicians after it is removed. An electronic module fabricated with common, inexpensive electronic components was encapsulated in a silicone intravaginal ring. The device uses temperature as a surrogate measure of periods of device insertion and removal, and stores a record of the data for subsequent retrieval. The adherence-monitoring intravaginal ring accurately recorded the device status over 33 simulated IN-OUT cycles and more than 1000 measurement cycles *in vitro*. Following initial *in vitro* testing in a temperature-controlled chamber, the device was evaluated *in vivo* in sheep using a predetermined insertion/removal pattern to simulate intravaginal ring use. After insertion into the vaginal cavity of a sheep, the logged data correctly indicated the device status over 29 hours of continuous measurement including three cycles of insertion and removal. The device described here is a promising, low-cost method for real-time adherence assessment in clinical trials involving medicated intravaginal rings or other intravaginal devices.

## Introduction

The ability to assess adherence to therapy in clinical trials is essential to accurately evaluate the efficacy of HIV infection prevention methods. Four recent clinical trials have demonstrated that pre-exposure prophylaxis (PrEP) regimens may prevent HIV infection in a significant proportion of individuals [1–5]; however, two additional HIV PrEP trials showed no efficacy [6, 7]. The trial failures were due in large part to poor adherence to the prescribed antiretroviral dosing regimens [8]. The importance of adherence in topical PrEP is underscored in the results of the CAPRISA 004 trial of a 1% tenofovir vaginal gel. The decrease in HIV infection was 54% in women with over 80% adherence to prescribed, pericoital gel use compared to 28% in women with less than 50% adherence [3]. Self-reporting adherence has been shown to be highly inaccurate: in the Carraguard microbicide trial, participants reported 94% gel use, but a dye indicator on the applicators showed only 61% of returned applicators had been used in 43% of sex acts [9]. Assessment of intravaginal ring (IVR) adherence is more difficult because a single IVR is often used for one month or more, and can be removed for long periods. The ASPIRE clinical trial of an IVR delivering dapivirine showed increased efficacy against HIV-1 infection in subgroups exhibiting the highest adherence; however, accurate assessment of continuous IVR use over 1 month was difficult using the metrics of amount of dapivirine recovered from used devices and plasma dapivirine concentrations at clinic visits [5].

Electronic devices have been used previously for measuring adherence to medication, and include electronic pill monitors and inhaler dose monitors. The costs and benefits of these devices have been reviewed extensively [10–16]. Drawbacks of existing technologies include high cost, reliability, and lack of correlation with therapy adherence, *i*.*e*. opening the pill bottle as indicated by a pill monitor does not indicate ingestion of the pill. Recently, Malcolm, *et al*. reported a method for measuring adherence to intravaginal implant use by monitoring vaginal temperature using a commercially available DST nano-T implantable temperature logger (Star-Oddi, Gardabaer, Iceland) embedded in the device [17]. The DST-nano-T devices encapsulated in silicone tubing of varying wall thickness to approximate an IVR accurately measured environmental temperature changes during 7 days of continuous monitoring *in vitro*. The devices also correctly recorded a series of insertion and removal events at 8 min intervals over 7 days of continuous use following vaginal insertion in three cynomolgus macaques. The cost of a single DST nano-T temperature logger (US$340), however, limits the potential of these devices to be widely adopted in large-scale clinical trials, particularly in resource-limited settings such as sub-Saharan Africa.

We report here an adherence monitoring IVR design that utilizes comparison of measured device temperature to a predetermined reference value to determine the insertion/removal status of the device. The custom electronic design is based on inexpensive, commercially available components, and a novel data reduction method allows for storage of up to 2048 status readings in only 256 bytes of non-volatile memory for retrieval after IVR removal.

## Materials and methods

### Device fabrication

**Fig 1:**
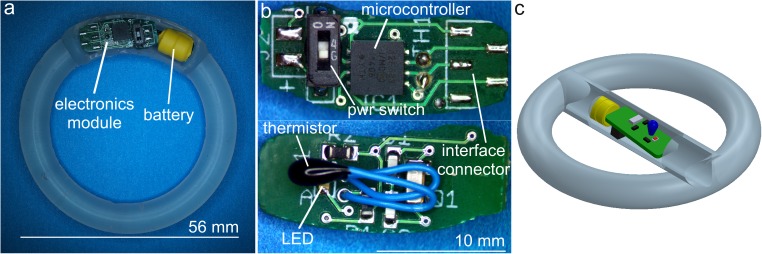
Adherence-monitoring IVR prototype. (a) Adherence IVR prototype with electronics module embedded in IVR elastomer circumference as used in *in vitro* simulation experiments. For clarity, the IVR is shown prior to sealing the circuit board and cylindrical battery pack in place using silicone adhesive. (b) Close-up of prototype electronics module. (c) Cutaway 3D model of adherence IVR prototype used in sheep study. The electronics module and battery are enclosed in a central compartment rather than sealed in the IVR circumference to allow access to the electronics during initial *in vivo* evaluation. A portion of the central compartment wall is removed in the drawing to expose the electronics module and battery.

The electronics modules were manufactured using industry standard printed circuit board (PCB) fabrication and surface-mount assembly methods, and consisted of a single *ca*. 5 mm × 12 mm PCB containing all electronic components including the thermistor temperature sensor and a separate battery pack containing two 1.55V SR416 silver oxide batteries (Fig 1). Electronic components were purchased from Digikey (Thief River Falls, MN, USA). The PCBs were fabricated by Sunstone Circuits (Mulino, OR, USA) and board assembly was completed by Screaming Circuits (Canby, OR, USA). Silicone IVRs with an open cavity for the electronics module were fabricated by injection molding from liquid silicone resin (MED-4840 LSR, Nusil, Carpenteria, CA, USA) using a custom single cavity mold and a laboratory-scale injection molding machine designed and constructed in-house [18]. Assembled electronics modules were sealed in the IVR using a silicone adhesive (MED3-4213, Nusil, Carpenteria, CA, USA) so that the electronics module and battery are completely encased in silicone and with no contact with vaginal fluid.

### In vitro evaluation

The performance of the adherence IVR was evaluated *in vitro* by exposing the devices to controlled temperatures simulating insertion and removal events. Devices were placed in a small aluminum enclosure with a flexible silicone heater (Watlow, St. Louis, MO, USA) and solid-state temperature controller (CT325, Minco, Minneapolis, MN, USA) providing accurate (±0.2°C) temperature control. The time, controller temperature set-point, and measured temperature were logged using a PC-based data acquisition system (National Instruments USB-6002 DAQ, Austin, TX, USA) using software written in the LabView development environment (National Instruments). For experiments spanning multiple days, the enclosure temperature was cycled between an “IN” state (~37°C) and “OUT” state (ambient, ~25°C) at predetermined intervals to simulate a sequence of IVR insertions and removals.

In order to determine the response time of the adherence monitor to temperature change, the thermistor voltage, V_th_ was measured as a function of ambient temperature as the device was cycled alternately between chambers held at 28°C and 37°C. Values of V_th_ were converted to temperature using a second-order polynomial fit to a five point temperature calibration curve over the range 25°C -44°C. The device response time was calculated as the time required to heat from 10% to 90% of the full-scale temperature difference (28.9°C to 36.1°C) or to cool from 90% to 10%.

### Simulated adherence monitoring in sheep

For *in vivo* evaluation in sheep, the adherence module was incorporated into a modified IVR design consisting of a standard silicone pod-IVR [18, 19] containing no drug pods with the module inserted into an additional hollow tubular compartment added to span the center of the ring. The central compartment configuration, rather than embedding the electronics module in the outer silicone ring, was used for the initial sheep study to allow facile access to the circuit components during testing. Devices were programmed, powered, and placed in Sleep mode, and then inserted into the central compartment and sealed with silicone adhesive.

The adherence-monitoring IVR was evaluated in a Rambouillet X Columbia sheep at the Colorado State University Preclinical Surgical Research Laboratory (Fort Collins, CO, USA). A schedule of ring insertion and removal was is shown in Table 1. The IVR was immersed in warm water bath (50°C) to initiate data logging, and remained in the warm bath until insertion in the sheep vaginal cavity. The IN/OUT status of the IVR was measured and stored in non-volatile memory on a 1.00 min interval, using a reference temperature of 34.4°C. The insertion/removal pattern was followed for 29 hours, whereupon the ring was removed and logging stopped by cutting through the ring to sever the battery connection to the PCB. The insertion and removal periods were based on the workflow in the animal facility during the two days of the study, and were designed to provide both short and long insertion and removal periods with minimal disturbance to the animal. The stored IN/OUT data stream was downloaded from the device to a PC using the Microchip PICkit3 programmer USB interface, and the data in hexadecimal format was converted to a series of binary (0 or 1) values indicating IVR OUT or IN.

**Table 1:** *In vivo* sheep study events timeline.

Time	IVR Status	Actual Temperature (°C)	Expected IVR Response*
**9:45 AM**	Logging Start, 50°C Bath	50°C	IN
**10:17 AM**	Insert in vaginal vault		
**1:11 PM**	Remove to 50°C bath		
**1:18 PM**	Remove to RT	25°C	OUT
**1:29 PM**	Insert in vaginal vault	41°C	IN
**3:35 PM**	Remove to RT	25°C	OUT
**3:52 PM**	Insert in vaginal vault	41°C	IN
**7:57 AM**	Remove to RT	25°C	OUT
**2:31 PM**	Stop Logging	25°C	none

RT, room temperature (~25°C)

* IN represents binary 1, OUT represents binary 0

The sheep study was performed according to NIH guidelines at Colorado State University, a facility accredited by the Association for Assessment and Accreditation of Laboratory Animal Care (AAALAC). Protocols were approved by Institutional Animal Care and Use Committees at Colorado State University (Fort Collins, CO, USA; Animal Welfare Assurance Number: A3572-01) under approval #12-3704A. Implantation of IVRs was performed under minimal physical restraint without causing undue stress or pain to the sheep. Sheep were flipped, hooded and hobbled, and placed in lateral recumbency. The perineal region was prepped with multiple scrubs of povidine iodine alternated with isopropyl alcohol. The device was inserted into the cranial vagina using a sterile gloved finger lubricated with medical-grade lubricant gel. A non-absorbable nylon suture tied to the IVR was allowed to extend outside of the vulva to indicate that the ring had not been expelled, and to aid in removal of the ring during removal/reinsertion procedures. Sheep were minimally restrained while the IVR was removed from the vagina using a suture traction. Devices were re-inserted using similar methods. The study was non-terminal.

## Results

### Hardware design

**Fig 2:**
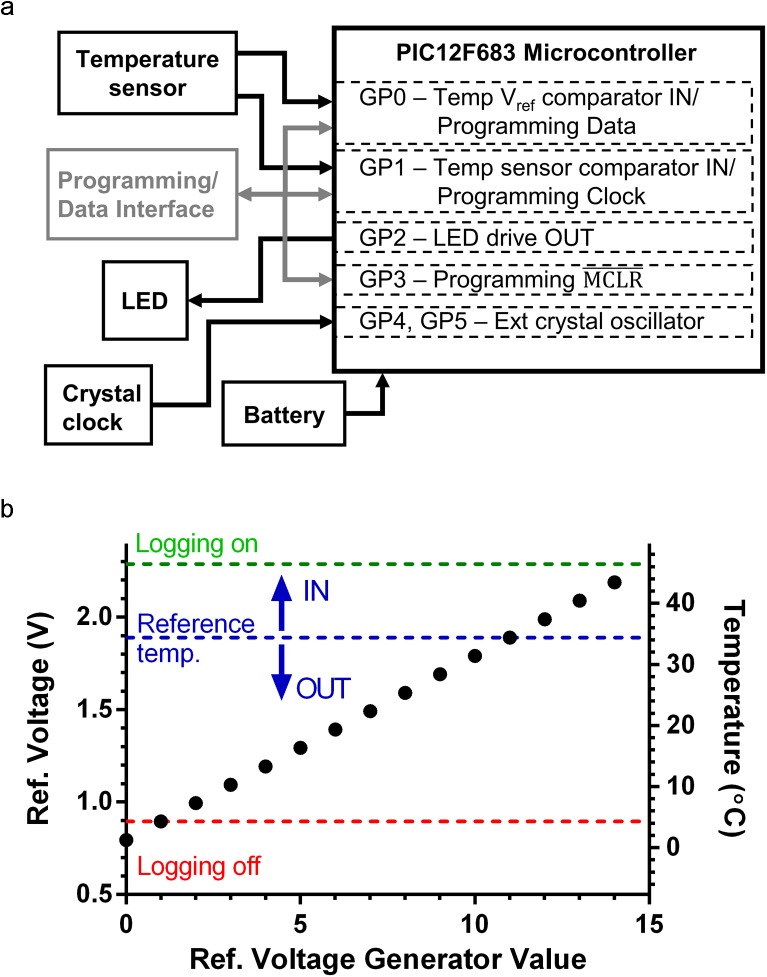
Electronics module design and function. (a) Diagram of adherence IVR electronics module showing major functional components (boxes) and dataflow (arrows). (b) Plot showing relationships between reference temperature, comparator reference voltage (V_ref_), and the 4-bit V_ref_ generator parameter. Each point represents a reference temperature that is set in software by the generator 4-bit value (0–15). Here a generator value of 11 is set to select a reference temperature of 34°C. Dotted lines indicate V_ref_ values selected for IN/OUT determination and “Logging On” “Logging Off” functionality in the *in vitro* and *in vivo* testing of adherence IVR devices.

The PIC12F683 microcontroller (Microchip Technology, Inc., Chandler, AZ, USA) was chosen as the basis of the adherence-monitoring IVR design. The PIC12F683 is a miniature 8-pin, Flash-based, 8-bit CMOS microcontroller, with a size of 4 mm × 4 mm × 0.9 mm in the DFN surface-mount package. The PIC12F683 has numerous features making it a good choice for this application, including: low-power sleep and operating modes to preserve battery life (50 nA standby current; 11 μA operating current at 32 kHz), analog comparator with on-chip programmable voltage reference, external oscillator functionality for precision long-term timing stability, six I/O pins individually programmable for sensor input or LED indicator output, and 256 byte non-volatile EEPROM memory capable of storing 2048 1-bit IN/OUT readings. The adherence monitor consists of the following functional modules (Fig 2A):

1. Battery: The device can operate with a voltage input range of 2.0 to 5.5 V. Two silver oxide coin cell batteries (SR416) provided a nominal 3.1 V input (V_dd_) voltage. For debugging and testing, the electronics module was powered by a variable DC power supply, and power for PIC12 microcontroller programming and data retrieval is provided through the programming/data interface connection.
2. Programming/Data Interface. A 5-pin interface is used for microcontroller programming and data transfer from the IVR to a PC for retrieval of logged status values. For the prototype device described here, all programming and data transfer operations utilized the Microchip PICkit 3 programming interface and the Microchip MPLabX Integrated Development Environment (IDE).
3. External crystal clock. A precision quartz crystal tuning fork provides a stable clock to drive all device timing. The crystal’s 32.768 kHz oscillations are counted into the PIC12 16-bit incrementing counter so that one timer rollover occurs every 2 s. This rollover initiates a wake from the microcontroller sleep state (wake every 2 sec), and all device timing (*e*.*g*. measurement interval) is a multiple of 16-bit timer cycles. The 200 ppm accuracy of the quartz crystal leads to timing accuracy of 6 ms h^-1^ (4.3 s per 30 days).
4. LED output. The general purpose I/O pin (GP2) is connected to an LED, allowing visual indication of device status, temperature measurement, and EEPROM writing. LED output is configured and enabled in software and allows for LED indicators in debugging mode, and the ability to turn off LED functionality programmatically to decrease device power consumption during long-term use.
5. Temperature sensor. A 10 kΩ precision thermistor temperature sensor in a resistive divider circuit provides a voltage input (V_th_) to the PIC12 comparator (GP1, CIN-) that is proportional to device temperature. The magnitude of V_th_ is a fraction of the battery voltage V_dd_ and varies linearly with thermistor temperature. The comparator compares this voltage to an internally generated reference voltage (V_ref_). Comparator output is high (1) when V_th_ > V_ref_ and low (0) when Vth < V_ref_. The value of V_ref_ is set programmatically with a 4-bit parameter, allowing a range of reference temperatures to be used for implementing various device functions such as starting or stopping data logging to internal memory.

### Device software

**Fig 3:**
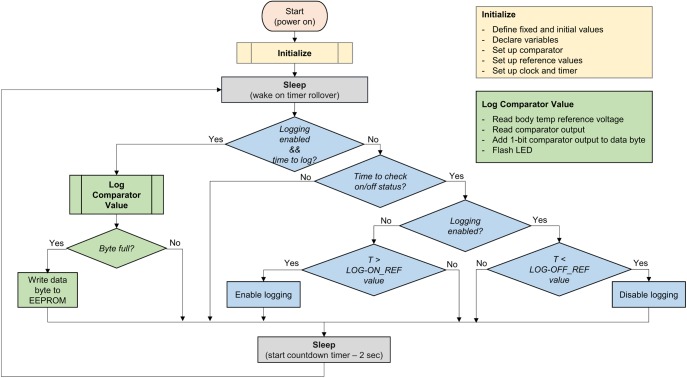
Flow diagram for adherence IVR embedded code. Major device program functions are indicated by color with arrows showing program execution flow: device initialization (orange), sleep state (gray), logging status control and timing (blue), and data logging and storage (green) functions.

The device’s embedded software was written in C using the Microchip XC8 compiler in the MPLAB X Integrated Development Environment (IDE). A code flow chart is shown in Fig 3. The compiled program is downloaded to the device using the PICkit 3 programmer. The software implements three main functions: measuring and saving to memory the 1-bit value representing device IN or OUT, enabling data logging, and disabling data logging. All are based on comparison of V_th_ to a V_ref_ generated in the microcontroller. Following completion of the initialization routine, the device enters a low-power sleep mode in a continuous loop waiting for 16-bit timer rollover. At each rollover (65538 counts at 32.768 kHz = 2 s), the device wakes and evaluates three options: (1) run the log comparator output (IN or OUT status) subroutine (green in Fig 3), (2) run the logging on/off subroutine (blue in Fig 3), or (3) return to sleep. The time interval for checking logging on/off status and logging comparator output is set in the program code. For prototype evaluation, the logging on/off subroutine was run every 2 rollovers (4 sec), and the data logging interval was varied from 10 s (5 wake cycles) to 5 min (150 wake cycles) depending on the test conditions.

Initially, a status bit indicating logging disabled prevents the execution of the logging (green) subroutine, and at wake the device queries if it is time to enter the logging on/off (blue) subroutine. If yes, the device measures V_th_ and compares it to the “Logging On” Vref value. The relationship between V_ref_ and temperature is shown in Fig 2B. If V_th_ > V_ref_ for a set number of wake cycles (typically 20 sec), data logging is enabled prior to returning to the sleep state. This has the effect of allowing data logging to be switched on by heating the device above the temperature corresponding to the “Logging On” V_ref_ value (typically 50°C). Once logging is enabled, subsequent wake cycles will run the green data logging subroutine at the appropriate sampling interval set in the program. When logging is enabled, each blue subroutine measures V_th_, compares it to the “Logging Off” V_ref_ (typically 5°C), and turns off logging if V_th_ < Vref. In typical real-world applications, the “Logging Off” functionality is not used and data logging is stopped by removing power (cutting the ring and battery connection).

When logging is enabled and the appropriate number of wake cycles have been completed to reach the set logging interval, the data logging (green) subroutine is entered. The program sets appropriate V_ref_ for determining IN/OUT status (typically ~35°C) at one comparator input and reads V_th_ at the other. The output is “1” of V_th_ > V_ref_ (IN) and “0” if V_th_ < V_ref_ (OUT). This 1-bit value is stored in the current data byte. When 8 IN/OUT bits are accumulated, the data byte is written to non-volatile EEPROM memory. The built-in PIC12F683 EEPROM size of 256 bytes allows storage of 2048 IN/OUT readings. Additionally, the LED may be flashed to indicate a measurement or other device status. The current implementation uses a short LED flash to indicate power on, a 3 sec LED on to indicate logging started or stopped, and a short flash to indicate a logged data value. More complex LED output enabled for debugging has included a double flash for “IN” measurement and single flash for “OUT” measurement with three flashes indicating a successful data write to EEPROM. The LED functionality may be enabled or disabled programmatically in software. During long-term testing and *in viv*o evaluation, the LED functionality is minimized to preserve battery life.

### Laboratory evaluation

**Fig 4:**
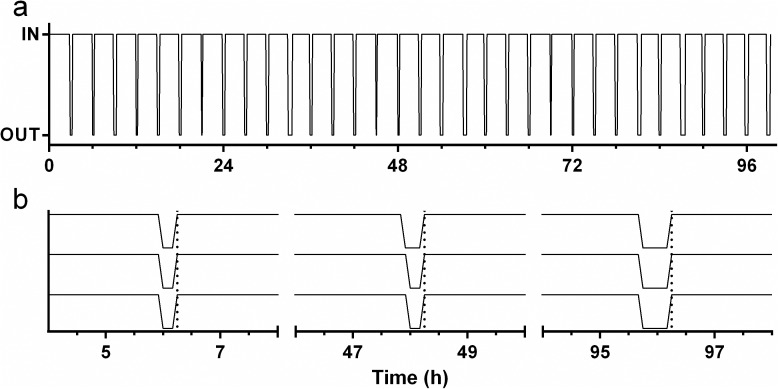
*In vitro* evaluation of adherence IVR. Response of adherence IVR over 100 h using a simulated insertion/removal cycle of 2.5 h in and 0.5 h out (lower plot) using a programmed temperature test apparatus. Data represent the IN/OUT status (0 or 1) logged at a 5 min interval. The upper plot shows expansion of data at 6, 48, and, 96 h for three adherence IVRs measuring simultaneously. Vertical dotted lines indicate transition to “IN” temperature for each cycle shown. The temporal alignment of “IN” measurements does not vary between the three devices over 96 h of measurement. The time required for the device to respond to a simulated insertion or removal event was determined using the temperature-time data shown in Fig 5. The device was exposed to three repeated step changes in temperature between 28°C and 37°C, and response was calculated as the time required for the device to measure the range of 10% to 90% of the total 9°C temperature change (28.9°C and 36.1°C). For the step from 28°C to 37°C (simulated insertion), the 10%-90% time was 1.84 ± 0.006 min. For the step from 37°C to 28°C (simulated removal), the 90% to 10% time was 1.96 ± 0.096 min.

The *in vitro* performance of an adherence-monitoring IVR undergoing multiple temperature cycles between 25°C and 40°C to simulate repeated insertion and removal over 100 h (4.2 days) is shown in Fig 4. The device accurately monitors the IVR status (temperature relative to 34.4°C) over 33 simulated IN/OUT cycles and 1192 measurements made on a 5 min interval. This is the equivalent of one IVR removal and replacement every 1.5 days for 50 days using a 1 h measurement cycle. The measurement frequency (1s –days) and reference temperatures (0–45° C) are set in software and cover a broad range of values appropriate for laboratory evaluation and long-term clinical use. The temporal accuracy of the device is shown in the expansion plot (Fig 4), where the simulated IVR replacements (temperature ~ 40°C) at approximately 6, 48, and 96 hours are captured at the same time by each of the three devices under test.

**Fig 5:**
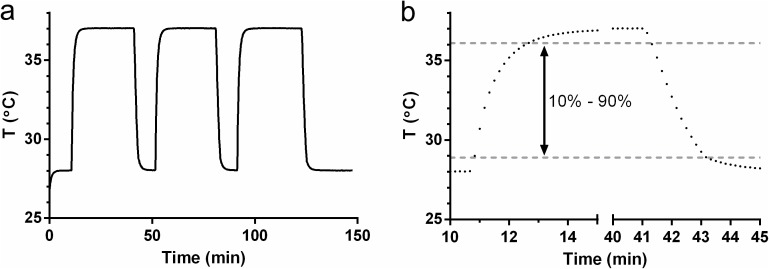
Adherence IVR temperature response analysis. (a) Temperature measured by the thermistor as a function of time during three cycles of alternating between two controlled temperature chambers maintained at 28°C and 37°C, respectively. (b) Zoomed data for first cycle showing individual temperature readings collected at 10 s intervals. The lower and upper gray dotted lines indicate the values at 10% and 90%, respectively, of the full-scale measured temperature change.

### Sheep study

**Fig 6:**
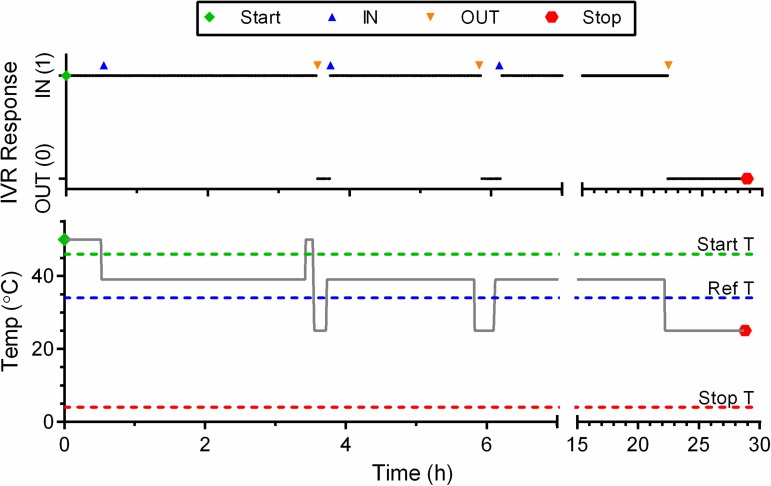
*In Vivo* Evaluation of Adherence IVR. Device response as a function of time for a series of insertion and removal periods in a sheep vaginal model. The black trace is the logged data (1 min logging interval) retrieved from the device at the study conclusion. The actual time for removal and insertion events is indicated by the symbols above the black trace. The gray trace shows the temperature as a function of time for the three ring dispositions: water bath (50°C) sheep vaginal temperature measured at the start of the study (39°C), and ambient laboratory temperature (25°C).

A single device was evaluated for 29 h over a series of three insertion/removal events in the vaginal cavity of a sheep. Fig 6 shows the IVR response to the sequence of events. The timing of the events is clearly captured by the device as shown by the alignment of the recorded data with the symbols indicating each insertion or removal.

## Discussion

The discordant results observed among HIV PrEP clinical trials clearly indicate the importance of adherence in determining successful prevention outcomes [8]. Sustained, topical delivery of ARV agents and microbicides using IVRs has been suggested for improving adherence; however, clinical data to support this assertion are limited [20, 21]. Real-time assessment of adherence during IVR clinical trials could allow quantitative measurement of the relationship between IVR use and HIV prevention efficacy. Until recently, no methods for real-time monitoring of IVR adherence had been reported. Boyd, et al. demonstrated proof-of-principle for using IVR temperature as a surrogate measure of adherence [17]. They employed a commercially available temperature logging device inserted in the vaginal cavity to continuously monitor device temperature, and compared it to intravaginal (body) temperature to determine periods of device insertion and removal. These devices store over 5000 temperature measurements for later retrieval and have a measurement accuracy of 0.2°C. The data collected during a 7 day macaque study using these devices embedded in segments of silicone tubing showed that device temperature can accurately track insertion and removal patterns even in the presence of normal vaginal temperature fluctuations. The $340 cost per device, however, limits the ability to incorporate these devices into IVRs for widespread use, particularly in resource-limited settings. Additionally, these are specialized devices from a single vendor, with a risk of limited availability due to market or other circumstances.

### Device design

The device described here offers a low-cost alternative for real-time temperature-based IVR adherence monitoring. The design is based on the widely used 8-bit Microchip PIC12 microcontroller platform. The circuit uses 8 discrete surface-mount components on a single PCB, with a total cost for components, PCB fabrication, and assembly of US$6 in quantities of 5000, including batteries. This is the cost for the device only and does not include IVR manufacture. A number of characteristics and advantages of the PIC12F683 microcontroller are described above. Use of the PIC12 internal comparator and non-volatile memory enabled the IVRs to use only 8 circuit components and achieve the smallest possible PCB size. The PIC12 data memory is limited to 256 bytes and required the use of a novel data reduction scheme to achieve storage of 2048 IVR status values for retrieval following IVR use. Our approach was to compare a measured voltage proportional to IVR temperature to a reference temperature to determine IVR insertion or removal status, but to store the data in bytes containing eight sequential 1-bit representations of OUT (0) or IN (1) instead of actual temperature values. Data files are retrieved from the device in hexadecimal format, and direct conversion to binary values results in a sequence of 0 and 1 values indicating IVR OUT or IN, respectively, at each predetermined logging interval. An important aspect of the device design is the ability to programmatically set the reference temperature through a simple software setting. For the studies described here, a reference temperature of 34.4°C was used. This temperature was selected based on the temporal variation in macaque vaginal temperature described by Boyd *et al*.[17] and the normal sheep body temperature measured in this study and by others,[22] with a margin for error to prevent false readings of IVR removal. The reference temperature will need to be optimized in clinical trial to accommodate differences between human and sheep basal temperatures.

A second device functionality that enables the simple, compact circuit design is the use of temperature readings to control device functions. As shown in the program flow diagram (Fig 3), data logging is started from the initial device sleep state when the measured thermistor voltage is above a reference value set programmatically. A reference corresponding to 50°C was used to initiate logging. Logging may be started by simply placing the device in a cup of hot water for approximately 1 minute. Water is used here as a convenience, and any method of raising the device temperature to 50°C or more would work equally well. Similarly, logging is stopped when the thermistor voltage is below a programmatically set “stop” threshold, easily achieved by exposing the device to a low temperature such as a cup of ice water or a refrigerator. A simpler method of stopping logging that may be more useful in a trial setting is to simply cut the IVR elastomer to sever the battery wires and remove device power. The stored data is retained in non-volatile memory in the unpowered device and may be retrieved through the program/data interface as described above.

A limitation of using temperature as a surrogate measure of adherence is that in some climates, temperatures may exceed the reference temperature, and even body temperature at times. Local temperatures at clinical sites used in the ASPIRE and Ring Study clinical trials of the dapivirine IVR are typically not high enough to be above the 34.4°C reference temperature used in these adherence IVR studies. Average high temperatures during the summer months approach this limit in some areas of South Africa and Zimbabwe, and high temperatures for individual summer days may exceed the reference or body temperatures. Issues associated with high local temperatures can be mitigated to allow retrieval of usable adherence data even in cases where ambient temperatures exceed the reference temperature. An IVR that is removed and stored at ambient temperature will record diurnal temperature variations if the daily ambient high temperature exceeds the reference temperature. It would be rare for the low temperature, even on the hottest days, to be above a 34–35°C reference temperature. This diurnal pattern will be distinct, and can be correlated with historical temperature records or temperatures measured at clinical sites as part of a trial protocol.

The adherence-monitoring IVR design is a modification of the established pod-IVR platform that was developed specifically for controlled vaginal delivery of drug combinations. The pod-IVR has been applied to delivery of small molecule ARV drugs for HIV [18, 19, 23–25] and HSV [18, 26] prevention as well as to vaginal delivery of monoclonal antibodies [27] and bacterial probiotics [28]. The pod-IVR consists of a silicone elastomer scaffold containing individual embedded drug pods that act as independent delivery devices. The adherence-monitoring module may be incorporated in the same manner as the drug pods, and drug release from the device will be unaffected. The dimensions of the prototype adherence module described here was chosen to be small enough to fit within a pod-IVR containing an expanded section as shown in Fig 1A, but large enough to allow manual soldering rework and other modifications during device development to be easily carried out. The electronics layout has subsequently been further miniaturized to a circuit board size of 14 mm × 5.1 mm that will fit within the dimensions of a standard pod-IVR (8 mm cross-sectional diameter, 56 mm outer ring diameter). This is also the size of the dapivirine IVR that has recently completed Phase 3 clinical trial [5]. Future efforts will focus on further miniaturization of the electronics module and the incorporation of adherence-monitoring functionality into pod-IVRs delivering ARV combinations.

### Implementation of adherence monitoring functionality in IVR designs

The *in vitro* and *in vivo* studies reported here demonstrate feasibility of the adherence monitoring approach and device; however, there are a number of development and regulatory hurdles associated with successful incorporation of the monitoring device in a combination product delivering one or more antiretroviral agents for HIV prophylaxis. The next steps in device development will focus on miniaturization of the electronics module and long term (≥28 days) *in vitro* and *in vivo* studies. The current devices are capable of this longer monitoring time. With a memory capacity of 2048 values, logging with a 30 min data interval allows continuous adherence monitoring for more than 42 days, and a 1 h interval increases this time to 85 days.

The regulatory pathway for a combination product consisting of an IVR delivering one or more antiretroviral agents modified with the adherence module is more complex than that of a drug or device alone. For incorporation of the adherence monitor into an IVR for HIV prevention, there are two primary regulatory concerns: (1) safety of the adherence module itself, and (2) demonstrating that addition of the adherence module does not negatively affect the safety, stability, or dreg release characteristics of the IVR. Because the primary mode of action (the mode expected to have the greatest overall therapeutic effect) of the combination device is antiviral drug delivery, the FDA Center for Drug Evaluation and Research (CDER) has regulatory jurisdiction, and clinical evaluation will take place under an Investigator New Drug (IND) application using a series of clinical trials (Phase 1, Phase 2, Phase 3).The adherence module is being developed primarily, but not exclusively, for incorporation into the pod-IVR platform that is currently in early clinical evaluation [26, 29]. Initial clinical evaluation of an adherence IVR to demonstrate feasibility would be conducted on the device without drug product under an Investigational Device Exemption (IDE) if the device is determined to be Significant Risk (SR). A SR designation for the adherence monitoring IVR is likely because it is an implantable device maintaining mucosal contact for more than 28 days. For initial clinical studies lasting fewer than 28 days, however, it is likely that the device would be designated Non-significant Risk (NSR), and evaluation could proceed without an IDE and only approval by the clinical investigator’s Institutional Review Board (IRB). This initial evaluation without drug product would provide a foundation of safety data to use in preparing the combination product IND as well as serve as Phase 1 clinical evaluation of an IVR that could be used in placebo groups in future clinical trials.

### Implications for clinical trial adherence monitoring

Adherence monitoring in HIV prevention trials has traditionally been limited to participant self-report or tracking of study materials (pill count or return of used gel applicators), approaches that have led to inaccurate assessment of true product use [8–10]. The ASPIRE dapivirine IVR trial incorporated an objective measure of adherence to IVR use in a large scale study in sub-Saharan Africa [30, 31]. Returned IVRs were tested for residual dapivirine levels to determine the amount of drug released. The distribution of residual dapivirine values was bimodal with one peak at 24–25 mg residual DPV indicating IVRs were unused, and a second peak at 20–21 mg indicating IVRs were used for 28 days. Using a length-adjusted analysis of the residual DPV data, seroconverting subjects were binned into four adherence groups based on the clinic visit with the lowest adherence for the three visits prior to HIV detection. A strong correlation of protection with adherence group was observed (% risk reduction, incidence per 100 person-years): no use (11%, 4.9), bottom third (29%, 3.1), middle third (58%, 1.9), top third (92%, 0.4). The placebo group exhibited an incidence of 4.7 per 100 person-years. Plasma levels of dapivirine was also measured quarterly as an additional measure of IVR use; however, insertion of the ring for 8 hours or more before a clinic visit resulted in identical plasma levels to 28 days of continuous use. Although the residual dapivirine levels only provided a snapshot of total IVR use over one month and no temporal use data, the ASPIRE data indicate that even imperfect and incomplete objective adherence data can lend valuable insight into patterns of adherence and the relationship between adherence and product efficacy in a clinical setting. The IVR use pattern obtained from the adherence module developed here would allow a much more detailed analysis than was conducted in ASPIRE, including specific time periods of IVR use, total drug dose during use period, self-report of IVR use and sexual activity, and HIV infection status to extract the key pharmacokinetic-pharmacodynamic relationships that result in HIV prevention efficacy.

Incorporation into a clinical trial can follow several approaches. (1) For a Phase III (efficacy) trial, all active and placebo IVRs can incorporate adherence devices. This will allow determination of adherence and investigation of product efficacy in both perfect adherence and non-adherence scenarios. Additionally, both placebo and active arms of trials can be monitored for adherence to determine patterns of adherence in different groups. Because residual dapivirine in returned IVRs and plasma dapivirine levels were surrogate measures of adherence in ASPIRE, no method for determining IVR use in placebo groups was available. Significant differences in adherence between active and placebo groups may indicate inadequate study blinding, poor randomization of study groups, or other trial design issues. (2) Adherence devices may be incorporated into a subset of Phase 3 study IVRs, both active and placebo. This will allow sub-group adherence data to be extrapolated to the larger study population in studies where it may be logistically difficult to include adherence monitoring for all study participants. Data from temperature-based adherence monitoring can also be combined with plasma drug levels and residual drug in used IVRs to provide temporal IVR use data to increase accuracy of adherence assessment. (3) Devices can be incorporated into IVRs in Phase 1 and Phase 2 trials as a “perfect” measure of adherence that can be used to evaluate non-objective adherence measures and to develop additional adherence endpoints, such as compounds adsorbed into the IVR from vaginal fluids, drug levels in blood or other relevant compartments, and residual drug in used IVRs.

## Conclusions

A miniature, low-cost IVR device using readily available electronic components, can provide accurate and quantitative determination of adherence to IVR-based HIV prevention methods. Adherence has been shown to be a primary factor in PrEP and microbicide clinical trial failures, and adherence monitoring may allow more accurate evaluation of product efficacy, identify factors influencing product use, and enable early interventions to ensure successful preventative therapy. Based on these initial *in vitro* and *in vivo* results, this adherence-monitoring IVR merits further development and evaluation in preclinical and clinical studies.

## Supporting information

S1 File Raw data for the *in vitro* controlled temperature sequence study (three devices evaluated simultaneously). Microsoft Excel 2013 format (.xlsx)
(XLSX)

S2 File Raw data for the *in vivo* sheep study (one device). Microsoft Excel 2013 format (.xlsx)
(XLSX)
